# Tipping point analysis for the between-arm correlation in an arm-based evidence synthesis

**DOI:** 10.1186/s12874-024-02263-w

**Published:** 2024-07-25

**Authors:** Wenshan Han, Zheng Wang, Mengli Xiao, Zhe He, Haitao Chu, Lifeng Lin

**Affiliations:** 1 Department of Statistics, Florida State University, Tallahassee, FL USA; 2 Department of Biostatistics and Research Decision Sciences, Merck & Co., Inc, Rahway, NJ USA; 3 Department of Biostatistics and Informatics, University of Colorado Anschutz Medical Campus, Aurora, CO USA; 4 School of Information, Florida State University, Tallahassee, FL USA; 5 Global Biometrics and Data Management, Pfizer Inc., New York, NY USA; 6 Division of Biostatistics and Health Data Science, School of Public Health, University of Minnesota, Minneapolis, MN USA; 7 Department of Epidemiology and Biostatistics, Mel and Enid Zuckerman College of Public Health, University of Arizona, Tucson, AZ USA

**Keywords:** Arm-based model, Correlation, Meta-analysis, Robustness, Single-arm study, Tipping point analysis

## Abstract

Systematic reviews and meta-analyses are essential tools in contemporary evidence-based medicine, synthesizing evidence from various sources to better inform clinical decision-making. However, the conclusions from different meta-analyses on the same topic can be discrepant, which has raised concerns about their reliability. One reason is that the result of a meta-analysis is sensitive to factors such as study inclusion/exclusion criteria and model assumptions. The arm-based meta-analysis model is growing in importance due to its advantage of including single-arm studies and historical controls with estimation efficiency and its flexibility in drawing conclusions with both marginal and conditional effect measures. Despite its benefits, the inference may heavily depend on the heterogeneity parameters that reflect design and model assumptions. This article aims to evaluate the robustness of meta-analyses using the arm-based model within a Bayesian framework. Specifically, we develop a tipping point analysis of the between-arm correlation parameter to assess the robustness of meta-analysis results. Additionally, we introduce some visualization tools to intuitively display its impact on meta-analysis results. We demonstrate the application of these tools in three real-world meta-analyses, one of which includes single-arm studies.

## Introduction

Systematic reviews play a crucial role in evaluating multiple studies on specific research topics, providing valuable evidence to support healthcare guidelines and decision-making. A key component of systematic reviews is meta-analysis, which quantitatively synthesizes evidence from different studies to improve statistical efficiency, reduce bias, and identify discrepancies among studies. A critical challenge in contemporary meta-analyses is their results’ sensitivity to data, model specifications, or inclusion/exclusion criteria; this challenge has prompted various forms of sensitivity analysis. For instance, the fragility index, initially proposed to evaluate the impact of modifications in event status on the statistical significance of clinical trials, has been extended to assess the results of meta-analyses and network meta-analyses of multiple treatment comparisons [1–3]. Within the Bayesian framework, the synthesized results, particularly concerning interval estimates, are heavily influenced by the selection of prior distributions [4, 5]. In addition, different meta-analysis models can yield divergent conclusions for medical decision-making. This issue has been illustrated by Cornell et al. [6], who demonstrated inconsistent pooled results using different estimation approaches on the same datasets.

In current meta-analysis practices, the most prevalent models are contrast-based models that focus on estimating treatment contrasts. These models typically pre-determine a comparative effect measure and then combine study-specific contrasts into a synthesized effect estimate. Different from contrast-based models, arm-based models include arm-level data [7], focus on estimating arm-specific parameters across studies, and use the estimates of arm-specific parameters to generate a variety of effect estimates for treatment comparisons. In this way, they provide information on the absolute effect of each arm rather than solely focusing on the comparisons between arms. Although arm-based models used to be questioned for breaking the randomization of individual clinical trials [8], studies demonstrated that arm-based models could effectively respect randomization by modeling correlations among treatment groups across trials [9]. In summary, contrast-based models assume exchangeable comparative effects across trials, while arm-based models assume exchangeable absolute effects. Comprehensive comparisons by White et al. [10] and Karahalios et al. [11] concluded that both models are valid tools for meta-analyses but are preferred under different assumptions.

Arm-based models have recently received increasing attention from the evidence synthesis community due to their advantages over contrast-based models. First, arm-based models offer flexibility without necessitating comparative effect measures from each study. It has improved efficiency by borrowing information from double-zero events, single-arm studies, and historical controls with an easier model fitting and interpretation than a contrast-based model [12]. Second, arm-based models can estimate absolute treatment effects from single-arm studies [12–14]. Single-arm trials, frequently used in phase I and II clinical trials, allow researchers to evaluate the safety and preliminary efficacy of a treatment in a small group of participants before proceeding to larger, randomized controlled trials. Thirdly, they can simultaneously estimate various types of effect measures (e.g., odds ratios [ORs], relative risks [RRs], and risk differences [RDs]) based on the estimates of arm-specific parameters (e.g., overall event probabilities). Moreover, arm-based models can also estimate conditional effects given baseline population characteristics [15], making it more feasible than contrast-based models to deliver population-specific synthesized results.

While the arm-based model has its benefits and promising applications, its results rely heavily on the correlation between the outcome of treatment arms, a heterogeneity parameter that accounts for randomization within individual clinical trials [9]. In this paper, we refer to it as the *between-arm correlation*, which may reflect assumptions of meta-analysis designs. Some works showed that different design assumptions led to conflicting conclusions from meta-analyses on the same topic [16, 17]. Moreover, the between-arm correlation indicates the extent of information borrowing when incorporating single-arm studies. As a meta-analysis commonly contains only a limited number of studies, the estimation of this between-arm correlation may be unstable, which implies different design assumptions and affects the validity of the results. Thus, it is critical to assess the impact of its estimate on the robustness of the synthesized conclusions from an arm-based meta-analysis.

This article proposes a set of new methods based on the concept of “tipping point” to address this problem, and the robustness of meta-analysis results is assessed in terms of both point estimates (magnitudes of treatment effects) and interval estimates (which informs whether the treatment likely differs from the control). The term “tipping point” is commonly used in missing data imputation and refers to a critical threshold at which the study conclusions change direction [18]. Specifically, tipping point analyses in missing data imputation evaluate the robustness of missing data assumptions by adding a successive shift parameter to overturn the conclusion [19]. These analyses are commonly required by regulatory agencies as a routine measure to address missing data issues in clinical trials. However, they remain less recognized within the community of evidence synthesis.

In the subsequent sections of this article, we provide the empirical distribution of between-arm correlations in real-world meta-analyses. Then, we review an arm-based meta-analysis model — bivariate generalized linear mixed effects model (BGLMM) — and highlight the critical role of the correlation parameter in arm-based meta-analyses. Next, we propose novel tipping point methods for assessing the robustness of meta-analytical estimates and visualizing the results. Then, we demonstrate the application of the proposed methods through three case studies, encompassing scenarios with and without single-arm studies. Finally, we conclude the article with discussions.

## Methods

### Empirical distribution of between-arm correlations

**Fig. 1:**
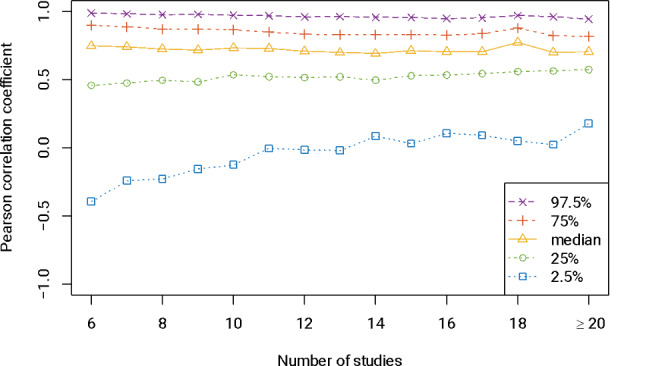
Percentiles of Pearson’s correlation coefficients between the observed event probabilities (on the logit scale) in the experimental and control groups among 69,133 Cochrane pairwise meta-analyses with binary outcomes

We draw on empirical evidence from a large collection of 69,133 meta-analyses from the Cochrane Database of Systematic Reviews to illustrate the importance of between-arm correlations in arm-based meta-analyses. This database has been utilized in our previous research on evaluating the empirical performance of meta-analysis methods, with the data collection process detailed therein [20]. Here, we included all Cochrane pairwise meta-analyses from 2003 Issue 1 to 2020 Issue 1, with binary outcomes that contain at least six studies. Their between-arm correlations can be illustrated by the correlations of observed event probabilities between two treatment groups. Figure 1 presents the percentiles of Pearson’s correlation coefficients among these Cochrane meta-analyses categorized by the number of studies. The median Pearson’s correlation coefficient was generally around 0.7. Although the distribution ranges typically shrunk as the number of studies increased, the ranges were still wide when meta-analyses contained quite large numbers of studies, with 2.5% and 97.5% quantiles being roughly 0 and 1, respectively. These observations indicate that between-study correlations are mostly positive, but they can be in a wide range, possibly affecting effect estimation.

### Arm-based meta-analysis model

#### Model specification

Suppose a meta-analysis contains $N$ studies. Without loss of generality, this article focuses on binary outcomes. The $i$th study has ${n}_{i1}$ subjects in the experimental group and ${n}_{i0}$ subjects in the control group ($i=1, 2, \dots , N$). Let ${\pi }_{ik}$ be the probability of events for subjects receiving the experimental ($k=1$) or the control ($k=0$) treatment in the $i$th study. The total number of events in the $i$th study’s group $k$ is ${X}_{ik}$, which is assumed to follow a binomial distribution $bin\left({n}_{ik},{\pi }_{ik}\right)$ [21].

We consider a BGLMM for the meta-analysis [7, 22, 23]; this model is referred to as bivariate because it assumes a bivariate normal distribution for the study-specific vector of transformed event probabilities for the control and experimental groups based on a specific link function:

$$\left(\begin{array}{c}g\left({\pi }_{i0}\right)\\ g\left({\pi }_{i1}\right)\end{array}\right) \sim N\left(\left(\begin{array}{c}{\mu }_{0}\\ {\mu }_{1}\end{array}\right), {\varvec{\Sigma }}_{\varvec{\mu }}\right), {\varvec{\Sigma }}_{\varvec{\mu }}=\left(\begin{array}{cc}{\sigma }_{0}^{2}& \rho {\sigma }_{0}{\sigma }_{1}\\ \rho {\sigma }_{0}{\sigma }_{1}& {\sigma }_{1}^{2}\end{array} \right).$$

Here, ${\sigma }_{0}$ and ${\sigma }_{1}$ are between-study standard deviations (SDs) of the two treatment groups, reflecting the heterogeneity in treatment arms across studies. The parameter $\rho$ accounts for the between-arm correlation, measuring the strength and direction of the relationship between treatment effects in two arms. It is possible to assume an equal between-study SD for both treatment groups (${\sigma }_{0}={\sigma }_{1}=\sigma$) to reduce the model complexity; this assumption can be assessed using criteria such as the deviance information criterion (DIC) [24] for Bayesian model selection. The link function $g\left(\cdot \right)$ has various choices, such as logit, probit, and complementary log-log transformation functions. In addition, ${\mu }_{0}$ and ${\mu }_{1}$ are the fixed effects of arm-specific event probabilities on the transformed scale.

When $g\left(\cdot \right)$ is the logit link function, $\text{e}\text{x}\text{p}({\mu }_{1}-{\mu }_{0})$ represents the conditional OR. The marginal event probability of treatment group $k$ can be approximated as ${\pi }_{k}=E\left({\pi }_{ik}\right)\approx \text{e}\text{x}\text{p}\text{i}\text{t}(\frac{{\mu }_{k}}{\sqrt{1+{C}^{2}{\sigma }_{k}^{2} }})$, where $C=16\sqrt{3}/\left(15\pi \right)$; here, the $\pi$ in $C$ represents the mathematical constant of about 3.14 [25]. It is smaller than the conditional event probability $\text{e}\text{x}\text{p}\text{i}\text{t}\left({\mu }_{k}\right)$, unless there is no heterogeneity among studies (${\sigma }_{k}^{2}=0)$, leading to equal conditional and marginal event probabilities. If we assume an equal between-study SD for the two treatment groups, the marginal OR, RR, and RD have expressions as OR $=\text{e}\text{x}\text{p}[\left({\mu }_{1}-{\mu }_{0}\right)/\sqrt{1+{C}^{2}{\sigma }^{2}}]$, RR $=\text{e}\text{x}\text{p}\text{i}\text{t}\left({\mu }_{1}/\sqrt{1+{C}^{2}{\sigma }^{2} }\right)/\text{e}\text{x}\text{p}\text{i}\text{t}\left({\mu }_{0}/\sqrt{1+{C}^{2}{\sigma }^{2} }\right)$, and RD $=\text{e}\text{x}\text{p}\text{i}\text{t}\left({\mu }_{1}/\sqrt{1+{C}^{2}{\sigma }^{2} }\right)-\text{e}\text{x}\text{p}\text{i}\text{t}\left({\mu }_{0}/\sqrt{1+{C}^{2}{\sigma }^{2} }\right)$. Based on the marginal event probabilities, the arm-based BGLMM can yield the marginal OR, RR, and RD [22, 26]. Interested readers may refer to McCullagh [27] for discussions on the differences in conditional and marginal inferences. In the following sections, we focus on the marginal inferences because all three commonly used effect measures (OR, RR, and RD) can be estimated under this framework.

### Model implementation

The BGLMM can be fitted using either frequentist [22, 28] or Bayesian approaches. The Bayesian approach is usually more computationally intensive than the frequentist approach, especially when conducting a more complicated network meta-analysis [29]. Nevertheless, it is less challenging for pairwise meta-analysis models, which are the focus of this article. We adopt the Bayesian framework, where parameters can be controlled through prior distributions, and use the R package “rjags” (version 4–13) [30] for all following analyses. We use the Markov chain Monte Carlo (MCMC) algorithm with three chains of different initial values, each containing 10,000 burn-in iterations among 100,000 iterations and a thinning rate of 2 to reduce auto-correlations. This provides 135,000 Gibbs samplers for each analysis, from which the treatment effects and correlation parameters can be estimated by the posterior medians and 95% credible intervals (CrIs).

### The influential role of the correlation parameter

The between-study variance-covariance matrix ${\varvec{\Sigma }}_{\varvec{\mu }}$ needs to be carefully estimated when fitting the BGLMM. Within it, the between-arm correlation is the key to account for randomizations within individual clinical trials in a meta-analysis. Inappropriate estimation of this parameter could affect the validity of conclusions from the arm-based meta-analyses. In addition, as discussed in Jackson et al. [31], there are two fitting approaches regarding whether to consider the uncertainty of the estimated variance-covariance matrix. The standard procedure approximates the true variance-covariance matrix with the estimated one when making inferences about the treatment effect. However, this approximation is improper when a meta-analysis includes a small number of studies. It remains unclear how many studies are needed in multivariate meta-analyses for reliable estimation of the variance-covariance matrix.

The Bayesian analyses allow for the uncertainty in the between-study variance-covariance matrix by placing priors on parameters, where external evidence can be incorporated via informative priors. However, researchers should be cautious when using weakly informative prior distribution because sometimes different priors can lead to markedly different results. For example, Wang et al. [5] evaluated the impact of covariance priors on arm-based meta-analyses and found that the commonly used conjugate inverse-Wishart (IW) prior distribution generally produces overestimation of variances and underestimation of correlations between treatment-specific log-odds. It can cause substantial bias in the estimation of log ORs and absolute effects. Other researchers also found considerable uncertainty in the between-study variance-covariance matrix estimation [32].

We simulated a dataset with 50 studies, each comprising 50 subjects in both the control and experimental groups (${n}_{i0}$=${n}_{i1}$=50). Table 1 illustrates the data structure for the first five studies. Simulation details are provided in Table 2. After randomly sorting these simulated studies, we conducted a set of meta-analyses using the first 5, 10, 15, 20, 30, and 50 studies sequentially. Table 2 indicates great uncertainties in estimating the between-arm correlation, with notably wide 95% CrIs persisting across different numbers of studies. For instance, the meta-analysis with five studies yielded 95% CrI ($-$0.382, 0.973) for $\rho$, which covers a wide range of the correlation’s possible domain ($-$1, 1). Even with 50 studies, the interval estimate is still wide, with 95% CrI (0.413, 0.807), indicating substantial uncertainty. Nevertheless, real-world meta-analyses often involve a limited number of studies [33, 34]. As shown in our examination of 69,133 pairwise meta-analyses from the previous Cochrane database, the median number of studies was 3. Furthermore, 89.7% of them included fewer than 10 studies, with only 2.5% involved more than 20 studies. Consequently, considerable uncertainties in the between-arm correlation estimates are common in real-world meta-analyses, posing a big challenge for valid arm-based meta-analyses. To address this, we proposed tipping point analyses to assess the robustness of meta-analysis results to the correlation parameter.

**Table 1:** The first five studies in a simulated meta-analysis with a binary outcome

Study ID $(i)$	No. of events in the control group $\left({X}_{i0}\right)$	No. of subjects in the control group $\left(n_{i0}\right)$	No. of events in the experimental group $\left({X}_{i1}\right)$	No. of subjects in the experimental group $\left(n_{i1}\right)$
Study 1	21	50	17	50
Study 2	9	50	17	50
Study 3	13	50	29	50
Study 4	9	50	15	50
Study 5	30	50	42	50

**Table 2:** True parameter values and their estimates in simulated meta-analyses. The true values of parameters for the simulation were $\rho =0.7$ (between-arm correlation), ${\sigma }_{0}={\sigma }_{1}=1$ (between-study standard deviations), and ${\mu }_{0}=0$, ${\mu }_{1}=0.5$ (event probabilities on the logit scale). The estimates were obtained using posterior medians with 95% credible intervals (Crls) in parentheses. The MCMC algorithm used three chains of 10,000 adaptation iterations, 100,000 iterations, 10,000 burn-in iterations, and a thinning rate of 2

No. of study	Marginal OR (95% CrI)	Marginal RR (95% CrI)	Marginal RD (95% CrI)	$\widehat{\rho }$ (95% CrI)	${\widehat{\sigma }}_{0}={\widehat{\sigma }}_{1}=\widehat{\sigma }$ (95% CrI)
True value	1.54	1.21	0.11	0.7	1
5	1.860 (0.746, 4.500)	1.430 (0.849, 2.450)	0.145 ($-$0.069, 0.345)	0.644 ($-$0.382, 0.973)	1.030 (0.559, 2.430)
10	1.900 (1.110, 3.190)	1.440 (1.060, 1.990)	0.153 (0.025, 0.273)	0.630 ($-$0.048, 0.925)	0.879 (0.582, 1.460)
15	1.870 (1.320, 2.680)	1.350 (1.140, 1.630)	0.153 (0.068, 0.239)	0.811 (0.423, 0.960)	0.974 (0.697, 1.460)
20	1.690 (1.160, 2.450)	1.260 (1.070, 1.520)	0.128 (0.036, 0.217)	0.659 (0.250, 0.874)	1.010 (0.764, 1.400)
30	1.700 (1.300, 2.210)	1.260 (1.120, 1.420)	0.128 (0.063, 0.192)	0.722 (0.432, 0.884)	0.940 (0.746, 1.230)
50	1.690 (1.350, 2.130)	1.250 (1.130, 1.390)	0.127 (0.073, 0.182)	0.649 (0.413, 0.807)	0.960 (0.803, 1.170)

### Tipping point analysis for the between-arm correlation

#### Tipping point analysis regarding interval estimates

Recall that through the implementation of BGLMM, estimates of comparative effect measures (e.g., OR, RR, and RD) can be obtained. The determination of an effect difference between two treatments relies on whether their interval estimates cover the null values (i.e., for OR and RR, the null value is 1; for RD, the null value is 0). In this article, we refer to it as an *interval conclusion*. In the preliminary Bayesian implementation of BGLMM, where the correlation parameter is assigned a weakly-informative prior distribution, we term the conclusion drawn from this approach as the *original* conclusion. To assess the robustness of these effect measure estimates regarding the original interval conclusion, a plausible range of values is assigned to the correlation parameter. The “tipping point” is identified as the value at which the original interval conclusion is flipped. This concept of a “tipping point” is borrowed from the sensitivity analysis of missing data analysis in randomized controlled trials.

For the arm-based meta-analysis model, we focus on investigating the tipping point for the between-arm correlation coefficient $\rho$. Potentially, $\rho$ can vary in a range ${R}_{\rho }$, with the most general case being ${R}_{\rho }$=($-$1, 1). Alternatively, one can incorporate clinical or statistical prior knowledge to restrict ${R}_{\rho }$ to a plausible range. For example, when both treatments are acknowledged to have effects in the same direction, one may only consider a positive correlation coefficient between the treatment groups. In this case, ${R}_{\rho }$=(0, 1).

As $\rho$ takes continuous values, we can simplify the implementation by discretizing ${R}_{\rho }$. Consider $B$ equally spaced points within the reasonable range ${R}_{\rho }$ (e.g., $B$=100 or by an increment of 0.01 in the $\rho$ value), we repeatedly estimate absolute and comparative effect measures with $\rho$ fixed at each value in the set of $\{{r}_{1}, {r}_{2}, \dots , {r}_{B}\}$. If the original interval conclusion is altered at $\rho ={r}_{b}$, where $b\in \{1, 2, \dots , B\}$, ${r}_{b}$ is identified as the tipping point and is denoted by ${r}_{b}^{tp}$. Due to Monte Carlo errors, the tipping point may not be a single point but may instead represent a range of values for $\rho$, within which the conclusion may oscillate before stabilizing. We define a set of all such tipping points as the “tipping range,” denoted by ${T}_{\rho }=\left[\text{min}\left({r}_{b}^{tp}\right),\text{max}\left({r}_{b}^{tp}\right)\right]$.

After obtaining the tipping point or the tipping range, we can compare it with the original estimates of the correlation, denoted as $\widehat{\rho }$, from the preliminary implementation. If tipping points are close to $\widehat{\rho }$, or if the tipping range contains $\widehat{\rho }$, the original conclusion can be easily flipped by changing the correlation coefficient, suggesting that the meta-analysis conclusion is not robust. On the opposite, if the tipping point or the tipping range does not exist or falls within an implausible region, the meta-analysis conclusion is robust. For example, if the tipping point of the correlation coefficient takes a negative value, but the two treatments are known to have a positive correlation, it suggests that the meta-analysis conclusion could still be robust.

### Tipping point analysis regarding point estimates

Monitoring the magnitude change of effect estimate is also important for assessing the potential impact of bias [35, 36]. Thus, we propose the tipping point analysis regarding point estimates in addition to the interval estimates. Similarly, the effect measure is estimated when the correlation parameter is assigned to plausible values. The tipping point or the tipping range is detected when the change of the estimated effect measure from its original estimate exceeds a pre-defined threshold. In this article, we consider the relative change, which is calculated as the new effect estimate (given a specific value for the correlation) minus the original effect estimate and then divided by the original effect estimate. The relative change can be positive or negative, implying the direction of changes in the new estimates. In the following examples, we use $\pm$15% and $\pm$30% as thresholds of low and high relative changes. Note that the threshold can be determined based on the clinical context from experts (e.g., clinically meaningful difference).

### Visualization of robustness assessment

We visualize tipping points regarding interval estimates in graphs by plotting both point and interval estimates of effects against the pre-specified range of the correlation coefficient values. We propose to use different colors to distinguish whether the assigned correlation coefficient value falls within the 95% Crl of the original correlation parameter and whether the conclusions of treatment effects are different from the original results. Specifically, the effect estimates are colored in black if the correlation coefficient takes values within the 95% CrI of its original estimate. The effect estimates are in blue when the correlation coefficient takes the value of its posterior median (i.e., the point estimate $\widehat{\rho }$). The effect estimates are in red when the interval conclusion is different from the original result (in terms of whether the CrI covers the null value). A transition to the red color suggests tipping points or tipping range of the correlation coefficient. If the potential tipping point is within the 95% CrI for the correlation coefficient, the effect estimates are colored in dark red. We also present the posterior density of the correlation parameter to reflect the likelihood of the tipping point or range. In summary, the plot suggests sensitive results to the between-arm correlation when several effect estimates are in dark red, and their corresponding tipping point values show high posterior density.

For tipping points regarding point estimates, we keep tracking the relative change in magnitude of estimated effect measures over assigned correlation values. The trajectory is colored in red if the assigned correlation value is within the original 95% CrI of $\rho$.

We will demonstrate these visualization approaches in the following section.

### Case studies

**Fig. 2:**
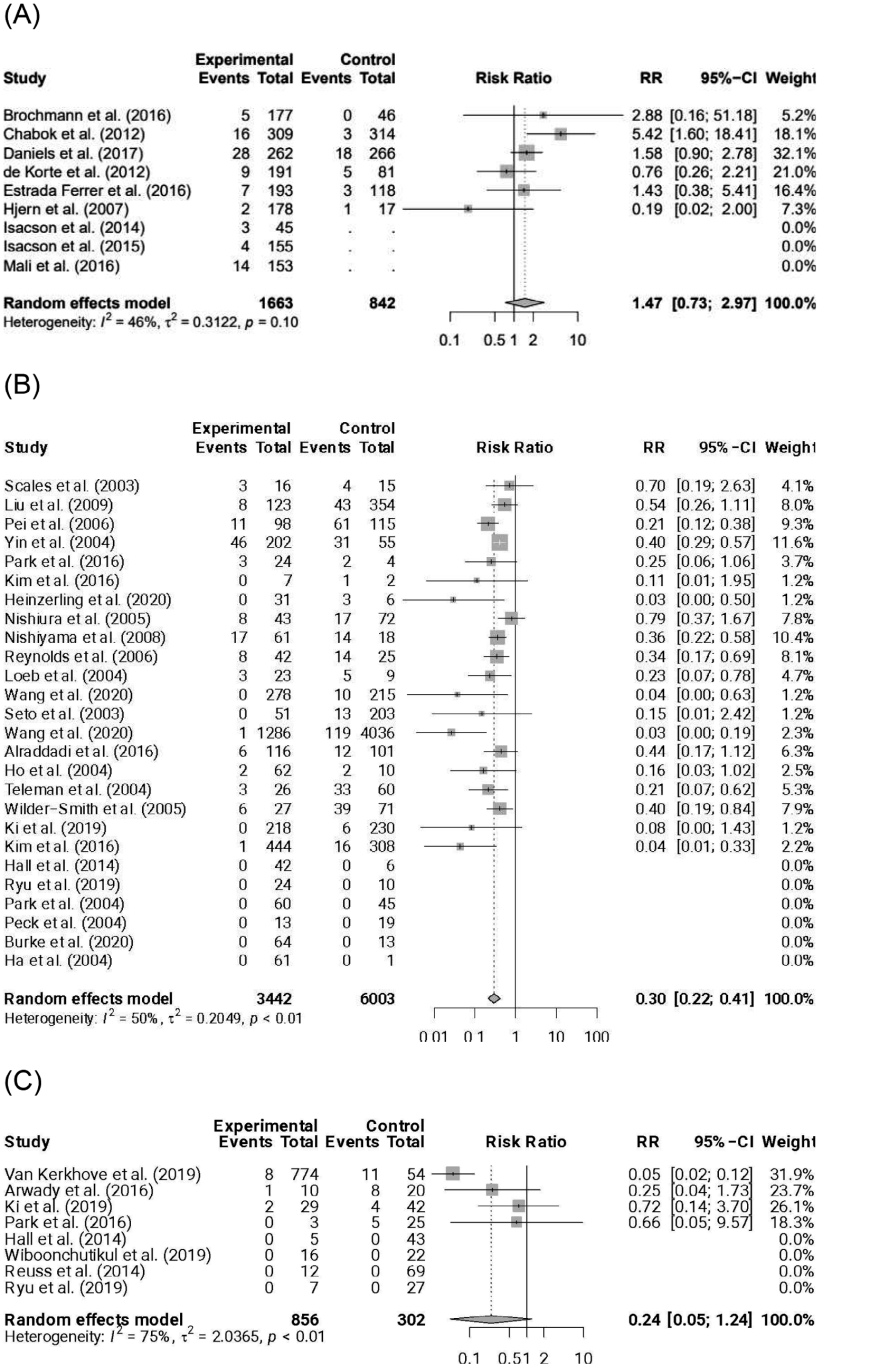
Forest plots for (**A**) the acute diverticulitis data, (**B**) the facial mask data, and (**C**) the physical distance data

We applied the proposed method to three pairwise meta-analyses with binary outcomes, comprising one with single-arm studies and two without single-arm studies. Figure 2 presents the forest plots of these three meta-analyses. The first meta-analysis by Au et al. [37] examined the risk of requiring additional treatment or intervention to settle in the initial episode for patients with uncomplicated acute diverticulitis. It compared patients receiving treatments without antibiotics to those receiving treatments with antibiotics. This meta-analysis had three single-arm studies exclusively focusing on treatments without antibiotics. Using a random-effects model would discard information from these three single-arm studies, resulting in a synthesized RR of 1.47, with a 95% confidence interval (CI) of (0.73, 2.97), based on the remaining six studies.

The other two meta-analyses are from the systematic review performed by Chu et al. [38], investigating the effects of preventive measures on virus transmission for respiratory diseases. One meta-analysis studied the effect of facial mask use on preventing respiratory disease infection in the healthcare setting. They obtained a synthesized RR of 0.30 with 95% CI (0.22, 0.41) from 26 studies using a random-effects model. Six studies were omitted from the analysis due to zero counts of infection events in both the experimental and control groups. Xiao et al. [39] re-analyzed the data with a frequentist BGLMM that accounted for double-zero-event studies and obtained the synthesized RR as 0.34 with 95% CI (0.23, 0.51). The last meta-analysis studied the effect of physical distance on preventing Middle East respiratory syndrome (MERS) with a synthesized RR of 0.24 and 95% CI (0.05, 1.24) in a random-effects model. In the original analysis, four studies did not contribute to the synthesized RR due to zero counts of infection events in both treatment groups.

We re-analyzed the foregoing three meta-analyses with the BGLMMs under the Bayesian framework with the logit link function. All parameters were assigned with weakly-informative priors: ${\mu }_{0}, {\mu }_{1} \sim N(0, {100}^{2})$, $\rho \sim U(-1, 1)$, and ${\sigma }_{0}, {\sigma }_{1} \sim U(0, 10)$.

**Fig. 3:**
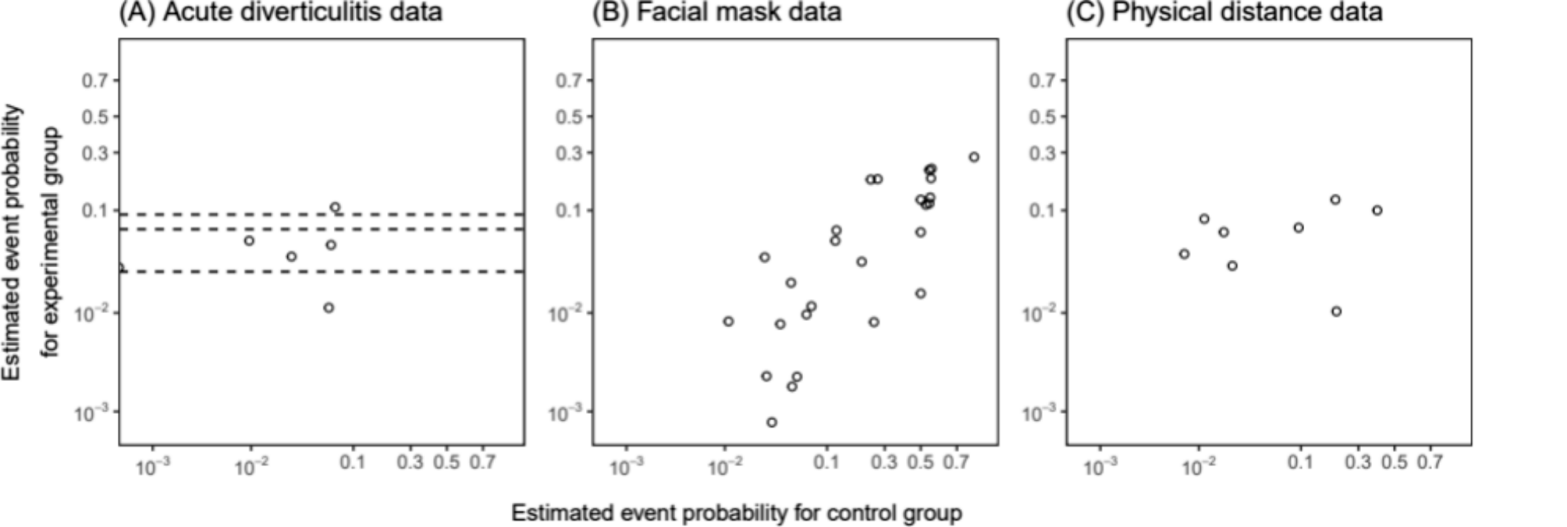
The scatterplots of event probabilities in the experimental and control groups for (**A**) the acute diverticulitis data, (**B**) the facial mask data, and (**C**) the physical distance data. Dashed lines in (**A**) represent event probabilities for single-arm studies. The 0.5 continuity correction has been applied to adjust for double-zero events in (**B**) and (**C**)

We first considered two candidate models with equal variance assumption (${\sigma }_{0}={\sigma }_{1}=\sigma )$ and unequal variance assumption (${\sigma }_{0}\ne {\sigma }_{1}$), and performed a model selection procedure to find the best model based on DIC with the *absolute difference* strategy, where we always select the less complex model if the difference in DIC is not greater than 3 [24]. We henceforth refer to the results selected in this step (rather than those initially reported in the source papers) as the original result of the meta-analysis. Our main interest is to assess the robustness of the original results. Table 3 presents these original results for the three meta-analyses. Figure 3 displays estimated event probabilities in the experimental group compared to the control group. It suggests a stronger positive correlation between the treatment groups in the facial mask data compared to the other two datasets. This evidence aligns with the estimates presented in Table 3, where the between-arm correlation coefficient $\rho$ for the facial mask data was around 0.9 with a narrow 95% CrI, while the other two datasets exhibited smaller correlation coefficients with wide 95% CrIs that included zero.

**Table 3:** Results of case studies based on posterior medians with 95% credible intervals (CrIs) under two model assumptions

	Equal standard deviation	Unequal standard deviations
**Meta-analysis 1: acute diverticulitis data**		
AR of control group	0.037 (0.019, 0.085)	0.049 (0.020, 0.240)
AR of experimental group	0.056 (0.035, 0.108)	0.057 (0.036, 0.114)
OR	1.560 (0.687, 3.370)	1.200 (0.197, 3.340)
RR	1.530 (0.702, 3.190)	1.190 (0.242, 3.150)
RD	0.019 ($-$0.020, 0.060)	0.009 ($-$0.177, 0.063)
Correlation $\rho$	0.415 ($-$0.555, 0.962)	0.376 ($-$0.609, 0.953)
${\sigma }_{0}$	0.726 (0.380, 1.420)	1.180 (0.396, 4.330)
${\sigma }_{1}$	0.726 (0.380, 1.420)	0.693 (0.317, 1.570)
DIC	77.14	78.10
**Meta-analysis 2: facial mask data**		
AR of control group	0.231 (0.152, 0.352)	0.227 (0.148, 0.337)
AR of experimental group	0.079 (0.044, 0.150)	0.094 (0.049, 0.183)
OR	0.290 (0.189, 0.423)	0.356 (0.221, 0.564)
RR	0.348 (0.235, 0.494)	0.417 (0.268, 0.634)
RD	$-$0.149 ($-$0.227, $-$0.095)	$-$0.129 ($-$0.198, $-$0.076)
Correlation $\rho$	0.916 (0.721, 0.982)	0.925 (0.750, 0.983)
${\sigma }_{0}$	2.030 (1.470, 2.970)	1.850 (1.310, 2.790)
${\sigma }_{1}$	2.030 (1.470, 2.970)	2.610 (1.750, 4.120)
DIC	212.86	209.70
**Meta-analysis 3: physical distance data**		
AR of control group	0.130 (0.043, 0.380)	0.149 (0.048, 0.399)
AR of experimental group	0.060 (0.012, 0.263)	0.044 (0.009, 0.235)
OR	0.428 (0.106, 1.630)	0.281 (0.037, 1.580)
RR	0.472 (0.125, 1.540)	0.319 (0.050, 1.480)
RD	$-$0.064 ($-$0.220, 0.051)	$-$0.091 ($-$0.319, 0.048)
Correlation $\rho$	0.738 ($-$0.309, 0.987)	0.620 ($-$0.672, 0.982)
${\sigma }_{0}$	3.180 (1.350, 8.330)	4.300 (1.650, 9.320)
${\sigma }_{1}$	3.180 (1.350, 8.330)	2.260 (0.009, 8.900)
DIC	44.30	44.33

DIC: deviance information criterion

Subsequently, the proposed method was applied with the selected model assumption to identify potential tipping points for the correlation coefficient $\rho$. We re-estimated a variety of effect measures, including treatment-specific absolute risks (ARs), ORs, RRs, and RDs, by setting $\rho$ to a range of values from $-$0.99 to 0.99 with an increment of 0.01. Due to space limits, we focus on tipping point analyses for $\rho$ regarding effect measures’ interval conclusions; the tipping point analyses regarding effect measures’ magnitudes are discussed only for the third meta-analysis.

All statistical code and data for implementing the proposed method and case studies are publicly available on the Open Science Framework (https://osf.io/8z9bp/).

## Results

### Acute diverticulitis data

**Fig. 4:**
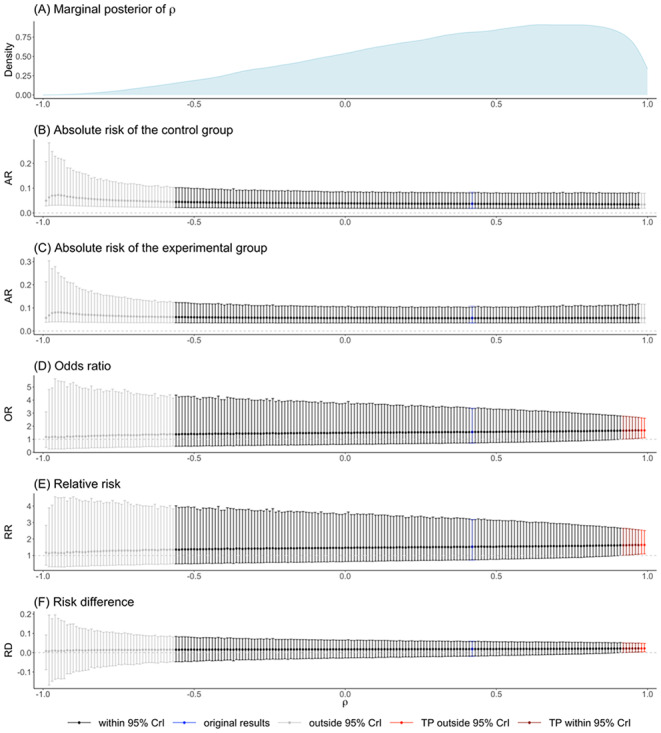
The estimated effect measures for the acute diverticulitis data when the correlation coefficient is fixed to values within ($-$1, 1) under equal standard deviation assumption ($\sigma_{0}=\sigma_{1}=\sigma$)

The DICs in Table 3 suggest the equal between-study SD assumption (${\sigma }_{0}={\sigma }_{1}=\sigma$) for the acute diverticulitis data. The posterior median of the correlation coefficient $\rho$ was 0.415, with a wide 95% CrI of ($-$0.555, 0.962). Figure 4 summarizes the results of our proposed tipping point analysis for $\rho$. In Fig. 4(A), the MCMC posterior density for the between-arm correlation $\rho$ is left-skewed. The comparative effect measures (i.e., OR, RR, and RD) showed increasing trends as assigned values of $\rho$ approaching one, indicating the effect of borrowing more homogenous information between the two treatment groups. Their corresponding interval estimates became narrower, which was expected due to increased information sharing between two treatment groups at such high correlations. Tipping points were observed at $\rho \ge$ 0.920, corresponding to 5.70% of $\widehat{\rho }$’s marginal posterior samples. This provides a quantitative measure of the uncertainty surrounding the original conclusion concerning the correlation parameter and the posterior probability of the tipping range of correlation. Therefore, evaluating the impact of tipping points should be complemented by analyzing the posterior density of the tested parameter. By integrating clinical insights regarding the desired precision in the results, one can determine whether the original conclusion is robust or not. The existence of a tipping point itself does not necessarily imply that the original results are not robust.

### Facial masks data

**Fig. 5:**
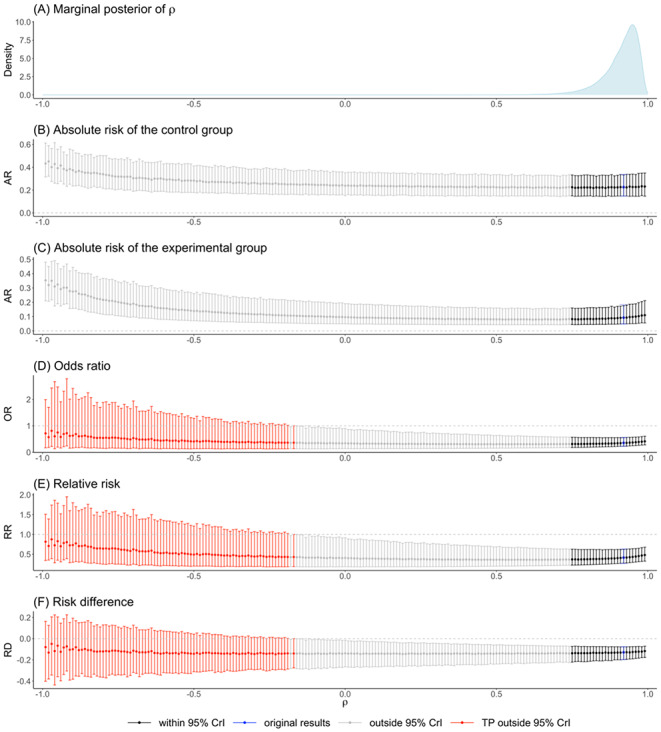
The estimated effect measures for the facial mask data when the correlation coefficient $\rho$ is fixed to values within ($-$1, 1) under unequal standard deviations assumption (${\sigma }_{0}\ne {\sigma }_{1}$)

In this case study, the model with heterogeneity SDs assumption (${\sigma }_{0}\ne {\sigma }_{1})$ was selected since the DIC difference between the two candidate models exceeded 3. The results of tipping point analyses are presented in Fig. 5. Overall, we found that the original results were robust to a wide range of values for the correlation coefficient. Although tipping points were observed, they fell outside of the 95% CrI for $\widehat{\rho }$, suggesting that the correlation coefficient was not likely to take values that could affect the conclusion. Based on these observations, we concluded that the effect estimates were stable and not sensitive to the variation of correlation values in the facial mask data.

### Physical distance data

**Fig. 6:**
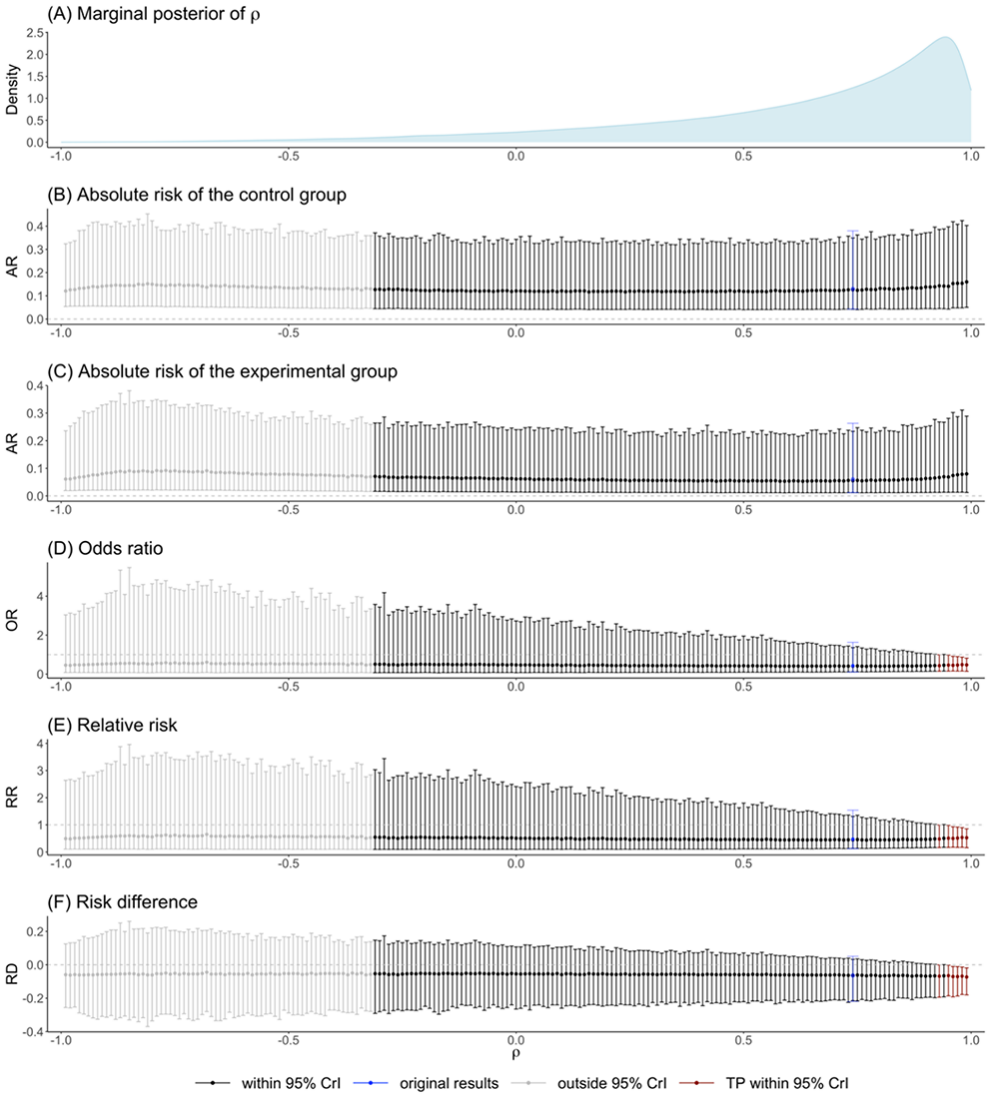
The estimated effect measures for the physical distance data when the correlation coefficient $\rho$ is fixed to values within ($-$1, 1) under equal standard deviation assumption (${\sigma }_{0}={\sigma }_{1}=\sigma$)

Based on the DICs, we assumed ${\sigma }_{0}={\sigma }_{1}=\sigma$. The results of tipping point analyses in Fig. 6 identified a tipping range of [0.93, 0.99] within 95% CrI of $\widehat{\rho }$. According to the posterior density, there were 17.3% of $\widehat{\rho }$ within this region. This raised concerns about the robustness of the original results regarding the correlation coefficient, as conclusions could be reversed due to a small shift in the correlation value.

**Fig. 7:**
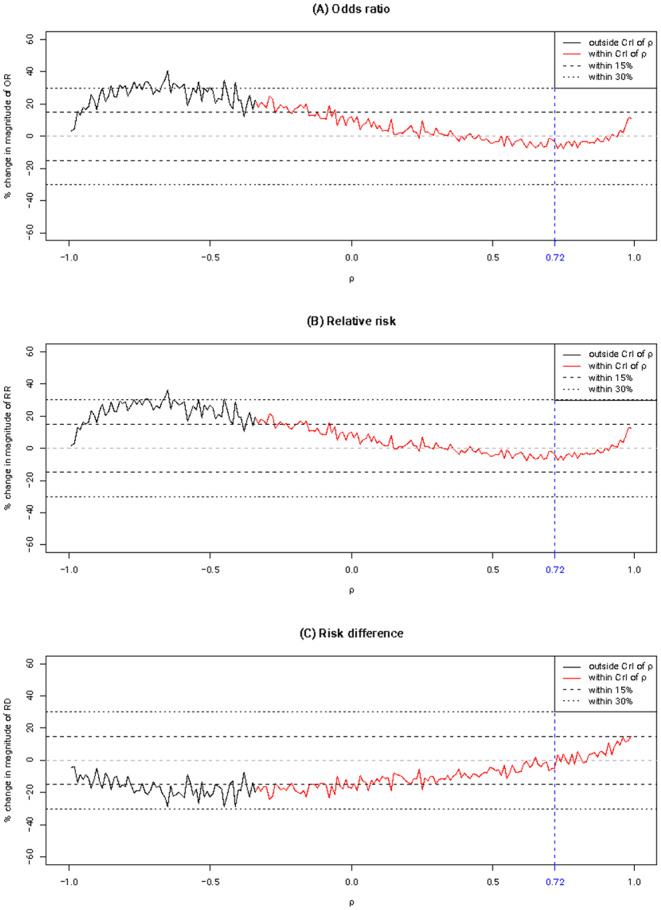
Tipping point analyses regarding effect measures’ magnitudes for the physical distance data

Figure 7 presents the tipping point analysis for $\rho$ regarding effect measures’ magnitudes. The solid lines show the trajectory of relative change in magnitude of effect measures (OR, RR, and RD) when $\rho$ is assigned to values between $-$1 and 1. For the values within the 95% CrI of $\widehat{\rho }$, the lines are colored in red; for those outside the 95% CrI of $\widehat{\rho }$, the lines are colored in black. The blue dashed vertical line marks the original point estimate of $\rho$. Overall, most parts of the red lines are within the 15% threshold, especially for those around the point estimate of $\rho$. This suggests that the point estimate of the correlation parameter is of less concern.

## Discussion

In meta-analyses without single-arm studies, fixing values of the correlation coefficient $\rho$ mainly impacts the interval estimates of effect measures. In meta-analyses with single-arm studies, non-robust results typically arise when most included single-arm studies have event probabilities deviating significantly from those of other comparative studies, as depicted by dashed lines in Fig. 3(A) but without intersecting the scatters. Therefore, our method provides an alternative approach to assess the reliability of results obtained from single-arm studies.

Previous studies in the literature primarily focused on the impact of prior distributions on Bayesian meta-analyses [4, 5], with little attention given to the impact of correlation parameters’ estimates on the meta-analysis results. Our study is the first to investigate such impact and develop novel methods to quantify and visualize it in the framework of Bayesian arm-based meta-analyses.

There are some limitations of this study. First, our analyses have mainly focused on interpreting the robustness of the results from a statistical perspective. However, clinical insights are highly needed in such an assessment, particularly when determining a reasonable range of values for the between-arm correlation coefficient. Second, fixing the correlation parameter to a specific value reduces the uncertainties in the estimates compared to the original analysis, where the correlation parameter is assigned to a prior distribution. Therefore, even assigning the correlation coefficient at the same value as its point estimate in the original analysis, the resulting estimates of effect measures could still be slightly different from the original analysis. Third, this article only considered the arm-based model. Contrast-based models are widely used in current meta-analysis research; similar approaches of tipping point analyses could be developed for contrast-based models.

In summary, sensitivity analyses are crucial for interpreting arm-based meta-analyses, which are of growing importance. Current sensitivity analyses often consider changes in events (such as the fragility index), model choices, and prior distributions used for Bayesian analyses. Our proposed tipping point analyses tackle the problem from a different perspective, considering the impact of correlation parameters on effect measure estimates. Future work could extend to other commonly used models in meta-analyses, such as the beta-binomial model [22] or contrast-based models. Contrast-based models do not involve between-arm correlations; their heterogeneity parameter is primarily the heterogeneity variance for a treatment contrast. The proposed tipping point analyses can also be extended to network meta-analyses, where multiple treatment comparisons can be jointly synthesized. Such an extension requires a more thorough consideration, as the variance-covariance matrix in this setting can become more complex.

## Conclusions

This article focused on the impact of the between-arm correlation on the results of arm-based meta-analyses, an increasingly useful method for including single-arm studies, historical controls, and population-specific estimates under the Bayesian framework. We have proposed a tipping point analysis method to quantitatively assess the robustness of meta-analysis results by assigning specific values to the correlation parameter within a plausible range. Innovative graphical tools have also been introduced to intuitively visualize the impact of the correlation parameter and its tipping points on the conclusions about treatment effects drawn from meta-analyses. We have demonstrated the proposed tipping point analysis on three real-world meta-analyses.

## Data Availability

The data that support the findings of this research are available from the forest plots in Fig. 2.

## Abbreviations

AR: Absolute risk
BGLMM: Bivariate generalized linear mixed effects model
CI: Confidence interval
CrI: Credible interval
DIC: Deviance information criterion
IW: Inverse-Wishart prior
MCMC: Markov chain Monte Carlo
MERS: Middle East respiratory syndrome
OR: Odds ratio
RD: Risk difference
RR: Relative risk
SD: Standard deviation
